# Semantic Modeling for Exposomics with Exploratory Evaluation in Clinical Context

**DOI:** 10.1155/2017/3818302

**Published:** 2017-08-30

**Authors:** Jung-wei Fan, Jianrong Li, Yves A. Lussier

**Affiliations:** ^1^Department of Medicine, The University of Arizona, Tucson, AZ, USA; ^2^BIO5 Institute, The University of Arizona, Tucson, AZ, USA; ^3^Center for Biomedical Informatics & Biostatistics, The University of Arizona, Tucson, AZ, USA; ^4^Cancer Center, The University of Arizona, Tucson, AZ, USA

## Abstract

Exposome is a critical dimension in the precision medicine paradigm. Effective representation of exposomics knowledge is instrumental to melding nongenetic factors into data analytics for clinical research. There is still limited work in (1) modeling exposome entities and relations with proper integration to mainstream ontologies and (2) systematically studying their presence in clinical context. Through selected ontological relations, we developed a template-driven approach to identifying exposome concepts from the Unified Medical Language System (UMLS). The derived concepts were evaluated in terms of literature coverage and the ability to assist in annotating clinical text. The generated semantic model represents rich domain knowledge about exposure events (454 pairs of relations between exposure and outcome). Additionally, a list of 5667 disorder concepts with microbial etiology was created for inferred pathogen exposures. The model consistently covered about 90% of PubMed literature on exposure-induced iatrogenic diseases over 10 years (2001–2010). The model contributed to the efficiency of exposome annotation in clinical text by filtering out 78% of irrelevant machine annotations. Analysis into 50 annotated discharge summaries helped advance our understanding of the exposome information in clinical text. This pilot study demonstrated feasibility of semiautomatically developing a useful semantic resource for exposomics.

## 1. Introduction

Precision medicine represents a paradigm of conducting biomedical research and practice by considering individual variation—genes, environment, and lifestyle [1]. Environmental and lifestyle factors play a critical role in our health and are known to interact with the genetic components through an epigenetic process [2]. The concept of exposome [3], which first came about in 2005, stands for all nongenetic factors that a person is exposed to throughout a lifetime. Common examples are pollutants, tobacco/alcohol use, occupational hazards, and even psychosocial stress such as being a victim of abuse. Exposome science has received increasing attention in precision medicine and has evolved into an interdisciplinary field across biology, genomics, public health, statistics, and informatics [4]. In order to capture the full picture of various health-related contexts, it is essential to incorporate exposome parameters into knowledge engineering and data analytics [5].

Whenever exposome is mentioned, common understanding refers to environmental conditions and self-induced exposures in social history. In fact, there has been a growing interest in using social history information (e.g., substance use and occupation) from clinical text [6–8]. On the other hand, often overlooked are exposures in clinical settings, many of which are the consequence of healthcare activities. Given the simplified equation “phenotype = genotype × environment,” extensive research has focused on phenotyping [9] from electronic health records (EHRs). Likewise, we propose that it is equally important to investigate all exposome information available in EHRs, a task dubbed as “expotyping” [10]. Dr. Vertosick Jr's quote, “You ain't never the same when the air hits your brain” [11] easily explains the implication that any procedure, especially a major one like craniotomy (plus the complication of anesthetics), may have a lingering effect on one's health (e.g., postoperative cognitive dysfunction [12]). In addition, research has revealed that even life-saving procedures could paradoxically result in an adverse outcome (e.g., ventilator-induced lung injury), depending on the subtle interplay with a patient's genetic predisposition [13]. Fortunately, modern EHRs can actually serve as a primary data source for tracing all types of clinical exposome on a patient. Another notable category is pathogen exposures, which often occur in disguise as diseases that can be deterministically attributed to a specific microbe. However, the challenge remains on how we can model and extract such abundant exposome information from EHRs.

Despite the diverse exposome data available in EHRs, there is still a dearth of systematic work on modeling and extraction. Possible explanations are (1) many healthcare activities are not perceived as exposures, (2) most research concentrate only on a narrow set of study-specific exposures, or (3) there is a lack of semantic resources available for assisting in the identification of exposome information in the EHRs. The most pertinent modeling framework we found was the Exposure Ontology (ExO) [14], which maps out key exposome concepts such as exposure event, stressor, and the relations among them. However, ExO is a bare skeleton that has not been filled with cross-references to concepts in other major terminologies and currently has limited coverage over the diverse clinical exposome of interest. To bridge these gaps with a focus on systematically investigating exposures in clinical contexts, we aimed to semiautomatically derive a semantic framework as well as explored its usability in facilitating exposome annotation of narrative EHRs. Our semantic modeling consisted of a subset of the Unified Medical Language System (UMLS) and, therefore, was interoperable with major biomedical terminologies. The interest in narrative EHRs was based on empirical knowledge that the texts not only serve as a good source of environmental/lifestyle factors but also document extensive clinical exposures (e.g., procedures). The annotation was meant to obtain general assessments on what exposome information is described in the clinical text, which would then lay the foundation for developing extraction tools and for discovering disease associations down the road.

The objectives of this study were (1) to create an exposome-oriented semantic network from existing ontology entities and relations, (2) to perform exploratory evaluation on the semantic adequacy and usability in clinical contexts, and (3) to summarize the properties of exposome concepts found in EHRs. In summary, our exposome subnetwork represented rich domain knowledge, including 454 pairs of exposure and outcome semantic types. The subnetwork concepts consistently covered about 90% of PubMed literature on exposure-induced iatrogenic diseases over a 10-year period. The subnetwork filtered out 78% of irrelevant machine outputs in the exposome annotation of 50 discharge summaries. Analysis of the annotations offers insights into the exposome presence in clinical contexts and feedback for existing ontology resources.

## 2. Materials and Methods

### 2.1. The UMLS and Semantic Network

The UMLS [15] is the world's largest integrated biomedical terminology framework maintained by the U.S. National Library of Medicine (NLM). The UMLS has three major components: the Metathesaurus (META), the Semantic Network (SN), and the Specialist LEXICON. The META unifies concepts from multiple source terminologies and ontologies into individual Concept Unique Identifiers (CUIs). As of the end of 2016, it contained more than three million concepts from 199 sources. The META includes files that host rich semantic information. The MRCONSO.RRF is the main file that collects comprehensive synonymous entities of the sources under each CUI. The MRSTY.RRF file links each CUI to their corresponding SN semantic type(s). The MRREL.RRF file preserves fine-grained semantic relations from source ontologies such as SNOMED-CT [16]. The SN currently contains 127 semantic types (e.g., T037 Injury or Poisoning) and 54 distinct semantic relations. The SN relations can be traced back to their origin from the NLM's Medical Subject Headings (MeSH) and are categorized as either hierarchical (*is-a*) or nonhierarchical (e.g., type A *causes* type B) [17]. For use cases that do not require fine granularity, there is also a mapping that aggregates the SN types into coarser semantic groups [18].

### 2.2. The i2b2 NLP Challenge Corpus

For studying the distribution and properties of exposome concepts in clinical texts, we used de-identified notes from the i2b2 NLP (Natural Language Processing) Research Data Sets that consist of medical records from institutions such as Partners HealthCare. With roughly a decade of history, the i2b2 challenges [19] facilitated clinical NLP research that varied from fundamental (e.g., coreference resolution) to end applications (e.g., identifying obesity). The 2006 corpus [20] for tasks of de-identification and smoking status classification was chosen for the current study—specifically a subset of Partners 889 raw discharge summaries without any annotation. The 2006 subset was chosen because the section headers occurred mostly with explicit uppercase patterns, making automated detection easier for computing section-wise distribution of the concept annotations. Further, the corpus had sentence boundaries predetected and therefore reduced the NLP effort. In a generalizability evaluation, we also used 73 independent discharge summaries from Beth Israel Deaconess Medical Center (or simply “Beth” hereafter), which was part of the i2b2 2010 corpus.

### 2.3. Extract Exposure-Related Semantic Types

**Figure 1:**
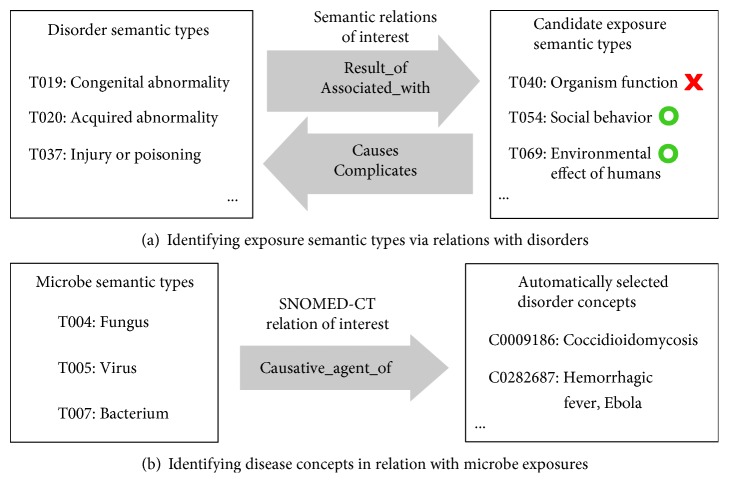
Ontology-assisted selection of exposome concepts.

To identify exposure-related semantic types, we started with disorder-related semantic types. This strategy was based on the assumption that any exposure event would generally result in a certain negative health effect.  Figure 1(a) illustrates our inference process using an event-driven template that has been framed into the question: What nongenetic factors could contribute to a disorder? The template was used to search the SRSTRE2 file of SN, which contained fully inherited relations among the semantic types. The disorder semantic types were obtained via the Disorder (DISO) of the SN semantic groups [18]. All the DISO semantic types were included except for T050 Experimental Model of Disease, which indicates an artificial setting. We manually determined four semantic relations that would involve a DISO type in an exposure (abbreviated as EXPO) event:

1. DISO is *result_of* EXPO.
2. DISO is *associated_with* EXPO.
3. EXPO *causes* DISO.
4. EXPO *complicates* DISO.

Many of the candidate EXPO semantic types matched the template; however, not all of them also fit into the context of an exposure event. For example, T019 Congenital Abnormality *associated_with* T040 Organism Function does not fit, as the latter is not a qualified EXPO type. Manual curation was performed over all candidate EXPO types suggested by the template, and only those that really meant an EXPO→DISO event were kept.

### 2.4. Extract Disease Concepts of Microbial Etiology

To identify microbe exposure where only the disease is mentioned, we used fine-grained semantic relations in the META MRREL.RRF file. The file contains semantic relations among UMLS concepts that were populated from the source ontologies. We reviewed all relations in MRREL.RRF and chose the *causative_agent_of* from SNOMED-CT to form a template.  Figure 1(b) illustrates our inference process of using the template. We manually determined three specific semantic types that represent the most common microbial exposures: T004 Fungus, T005 Virus, and T007 Bacterium. Searching with the template is equivalent to asking “the microbe concept is a *causative_agent_of* what?” The outcome slot is simply filled by selecting any concept that belongs to the DISO semantic group as described in the previous section. As a result, each identified disorder actually stands for “ever being exposed to” a responsible microbe, based on the domain knowledge modeled in SNOMED-CT.

### 2.5. Evaluate the Semantic Adequacy for Covering Clinical Exposome Literature

To evaluate if the semantic modeling reasonably covered domain knowledge on clinical exposome, we used PubMed literature about exposure-induced iatrogenic diseases during 2001–2010. The range to 2010 was used because PubMed records of the most recent years might be still being indexed and not reflect a stabilized view for the bibliometric purpose. The benchmark set consisted of 1248 PubMed IDs (PMIDs) from the query: “adverse effects”[sh] AND iatrogenic[ti] AND (“2001/01/01”[PDAT]: “2010/12/31”[PDAT]). The MeSH terms of the PMIDs were automatically extracted and mapped to CUIs using the MRCONSO.RRF source and synonym information. Given the CUIs of the MeSH terms, we were able to compute which MeSH terms belonged to the exposure or disease semantic types of our derived exposome subnetwork. Note that for a MeSH to qualify as an exposure, we also required it to be followed by the subheading “adverse effects” or any of its children “toxicity” and “poisoning.” For a PMID to be counted as being covered by the subnetwork, at least one MeSH had to belong to an exposure semantic type (plus proper subheading) and at least one MeSH belonging to a disease semantic type as in one of the covered EXPO-DISO relations (refer to Supplement 1 available online at https://doi.org/10.1155/2017/3818302). The chronological trends were summarized for the number of covered PMIDs and exposure-disease pairs, as well as the percentage of covered PMIDs and the average number of exposure-disease pairs per PMID.

### 2.6. Perform Exposome Annotation and Analysis in Clinical Text

For batch preannotation, the MetaMap [21] program (2016 version) was used to identify UMLS concepts in the 889 Partners discharge summaries. From the MetaMap-identified concepts, we filtered by keeping only those either with an exposure semantic type or of a microbe-caused disorder. In addition, regular expression (uppercase phrase followed by colon) was used to mark the section headers for calculation of section-wise distribution of the concepts. To obtain a more reliable summary of the exposome annotations, the first author (Ph.D. in biomedical informatics) manually curated the machine-identified candidate concepts in 50 random discharge summaries that had an explicit social history section. In terms of size, this subcorpus had an average of 115 sentences (or 1124 tokens) per document, and the average sentence length was 9.5 tokens. The curation involved verifying exposures that were present to the patient and correcting any mislabeled sections. The process was facilitated by using the *brat* annotation tool [22]. Based on the curated annotations, we calculated descriptive statistics of the concept distribution over different sections and semantic types. As a rough assessment of reproducibility, we used the curated exposure annotations to (case-insensitively) exact-match into 73 independent Beth discharge summaries and compared the distribution of sections that contained those exposures. Lastly, qualitative analysis was performed to understand characteristics of the exposome concepts and limitations of the existing ontology resources.

## 3. Results and Discussion

### 3.1. Modeling of Exposome Semantic Types and Concepts

**Figure 2:**
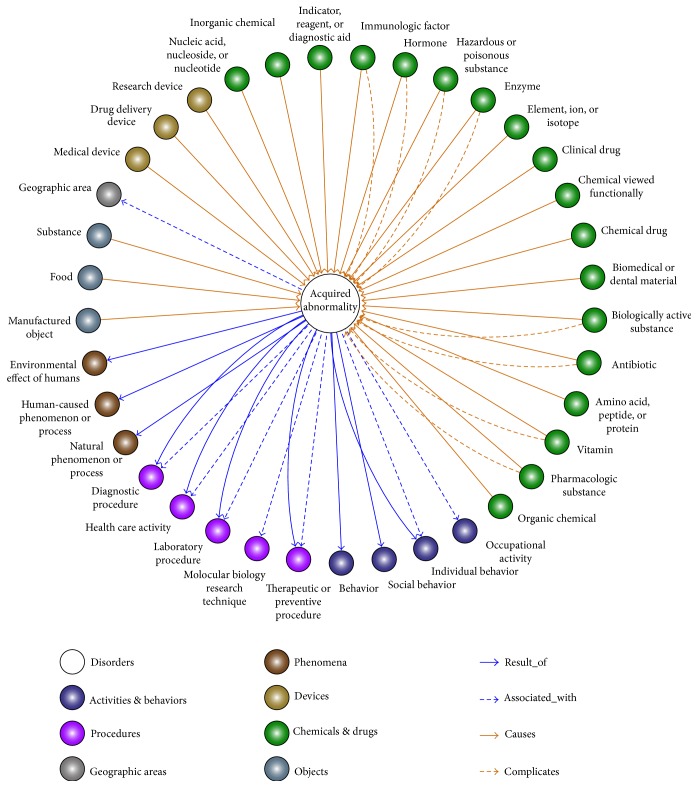
A partial network of exposome semantic types and their relations to health outcome. ^∗^The colors of the exposure nodes stand for different broad semantic groups that the semantic types belong to.

The methods of Figure 1(a) obtained 454 EXPO-DISO pairs that were linked via the relations *causes*, *complicates*, *associated_with*, and *results_of*. There were 41 distinct exposure semantic types involved in these relations. The full list of the curated semantic type pairs is available in Supplement 1. The EXPO-DISO pairs represent a comprehensive network of semantic types involved in exposure events. To provide a peek into the model contents, Figure 2 visualizes a partial network showing only exposure semantic types that are directly related to T020 Acquired Abnormality. Pointing outward are the relations *result_of* and *associated_with*, for example, Acquired Abnormality as *result_of* Therapeutic or Preventive Procedure. Inversely, the exposure nodes point to the center with the relations *causes* and *complicates*, for example, Pharmacologic Substance *causes* Acquired Abnormality. We color-coded the exposure nodes according to their broader UMLS semantic groups. It can be seen that Chemicals & Drugs (in green) dominate about half of the types, followed by Procedures (in purple), and then Activities & Behaviors (in indigo). The methods described in Figure 1(b) identified 5667 microbe-induced disorder concepts. Examples are shown in Table 1, and the full list is available in Supplement 2.

The results demonstrate rich domain knowledge that can be extracted from existing ontology. The derived subnetwork models comprehensive interactions involved in exposure events. One advantage of using the UMLS is that it allows linking the semantic framework to individual biomedical concepts and their source terminologies for versatile integration. The application of our methods offers great potential for enriching existing resources such as the ExO, which provides a core skeleton but lacks the integration with concepts of major terminologies. Our deliverable of the microbial disorder concepts can serve as a useful resource itself, especially for use cases dealing with infectious diseases. In the list, we also observed an interesting disorder, C0014522 Epidermodysplasia verruciformis, which is an autosomal recessive inherited skin disease that features wart-like lesion due to infection with the human papillomavirus (HPV). It echoes the importance of investigating subtle interplay between our genome and exposome in order to fully understand certain health conditions. In terms of methodology, our semantic filtering based on relational template demonstrates a useful ontological approach for selecting task-specific entities of interest.

### 3.2. Semantic Adequacy of the Derived Exposome Subnetwork

**Figure 3:**
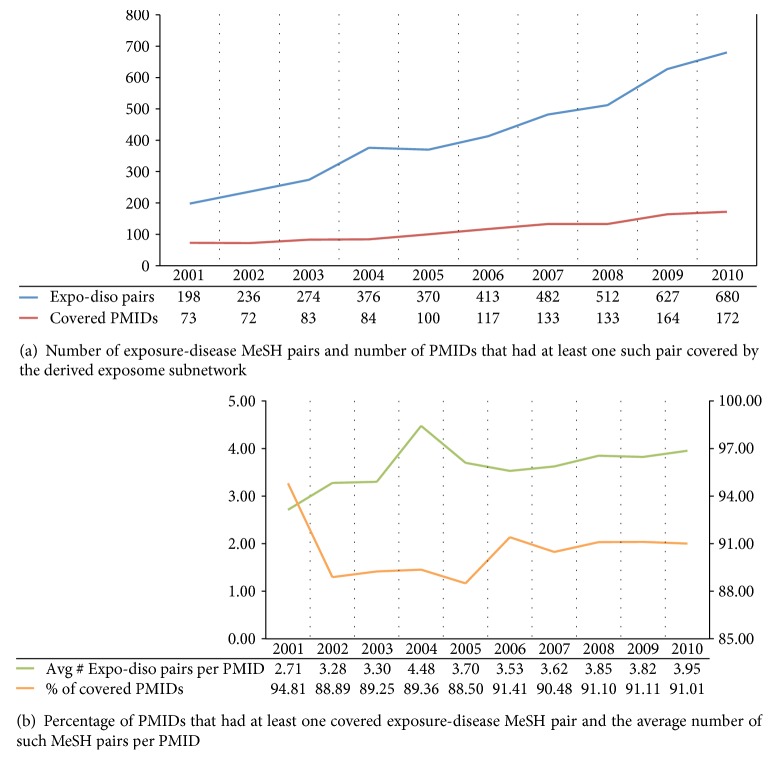
Semantic coverage of exposure-induced iatrogenic diseases in PubMed from 2001 to 2010.

For assessing whether our semantic framework adequately accommodates evolving evidence-based medicine, Figure 3 shows the trends of covered literature on exposure-induced iatrogenic diseases—which represent the primary area of interest in clinical exposome. In Figure 3(a), the red line indicates a steady increase of PMIDs that had at least one exposure-disease MeSH pair covered by our subnetwork. In terms of the distinct exposure-disease pairs, the blue line indicates corresponding growth over the 10 years.  Table 2 provides actual examples of the covered MeSH pairs and their host PMIDs. More importantly, not only do the counts indicate the coverage scales with the knowledge growth but the orange line in Figure 3(b) indicates that the percentage of PMIDs with covered exposure-disease pairs remained consistently high (mean = 90.59%, standard deviation = 0.02%). The green line shows that the average number of covered exposure-disease pairs per PMID climbed mildly over the years. As for the peak in the green line, it is unclear why in 2004 there was a sudden surge of research findings. Despite marginal errors in the bibliometrics-oriented evaluation, we believe it reasonably corroborates the reliability and scalability of our semantic modeling. In addition, since our subnetwork is derived from the UMLS, any update in the SN (though infrequent) can be incorporated on a regular basis.

### 3.3. Exposome Annotations in the Clinical Text

The filter of exposome semantic types and concepts consistently reduced the number of entities required for manual review. Specifically for the 50 random discharge summaries, an average of 77.72% presumably irrelevant (i.e., nonexposome) MetaMap annotations was filtered out. After the manual curation, the median number of annotations per note was 36 (min: 9, max: 118). According to the data use agreement, we will contribute our annotations back to the i2b2 clinical NLP repository. By aggregating counts across the notes, the top 20 sections with the curated exposome annotations are shown in Table 3. Due to the inpatient context of discharge summaries, the top section turned out to be HOSPITAL COURSE, which essentially covers all procedures and medications administered during a patient's hospitalization. Aligning with expectation, SOCIAL HISTORY ranked moderately high (the 8th) in the list. There were six medication-related sections in the top 20, which reflected drugs being a preeminent exposure in a clinical setting. The sections HISTORY OF PRESENT ILLNESS (the 2nd) and PAST MEDICAL HISTORY (the 7th) also hosted a decent amount of exposome annotations. As the assessment of generalizability across independent institution/dataset, Table 4 shows that we not only found many of the identical exposure terms in the Beth corpus (the column “# annotations” was based on exact-string search) but the sections hosting those terms also exhibit high similarity in terms of name and rank (comparing to Table 3). For example, the top two sections, HOSPITAL COURSE and HISTORY OF PRESENT ILLNESS, are identical. In Table 5, we list the top 20 semantic types of the curated concepts. The top two types, T121 Pharmacologic Substance and T109 Organic Chemical, are from drugs, consistent with the high prevalence of medication-related sections as in Table 3. The second cluster consists of T061 Therapeutic or Preventive Procedure and T060 Diagnostic Procedure, with most of them mentioned in HOSPITAL COURSE (not shown in the table). The type T055 Individual Behavior (e.g., use of alcohol or tobacco) mostly resides in SOCIAL HISTORY. For further inspection, Supplement 3 includes the details of the annotation counts ranked in two levels: first by section and then by semantic type.

The results show that our exposome semantic modeling helped prefilter about 78% of irrelevant concepts before the manual curation. Our attention to microbe-based exposures was justified: Although minor in proportion, the two types, T047 Disease or Syndrome (those of microbial etiology) and T007 Bacterium, made it into the second half of Table 5 (the 11th and 15th, resp.). Medications were found to form the majority of the clinical exposome, which echoes a previous work that also presented drugs as an “environmental” factor in EHRs [23]. Given the exposures predominantly being medications and procedures, one could argue that using structured EHR data would save the redundant effort of extracting them from text. However, it requires further investigation to understand how much data would be missed if using only structured data. For example, a previous study showed that provider notes are complementary to structured data for recording of medication intensification [24] and that we should take into account any over-the-counter products documented in history narratives. It is noteworthy that HISTORY OF PRESENT ILLNESS and PAST MEDICAL HISTORY ranked among the top 10 sections in Table 3, suggesting that the history sections that are generally included in most clinical notes (not just discharge summary) can host abundant exposome information. Among the exposome-containing sections, SOCIAL HISTORY should be considered as different by nature, for it distinctly covers most of the “nonclinical” exposomes encountered in a patient's daily life. In one specific note, the social history even documented exposure to a nuclear accident in the patient's past residence.

A pertinent question that emerged in the study was “to what extent could we generalize the definition of exposome?” We annotated concepts such as retirement and widowhood (both T033 Finding) as exposures, given that studies have shown their association with health [25, 26]. However, would it make sense to also treat any significant biological event such as pregnancy as an exposure? This remains an open subject of future research. Interestingly, an infrequent category of exposures revealed one blind spot of our semantic modeling, which could partially be attributed to limitations in the UMLS semantic classification. We came across several exposure concepts such as C3496069 Cocaine Use and C0206073 Domestic Violence, which were classified to type T048 Mental or Behavioral Dysfunction. However, the filter criteria missed them because we did not consider T048 to be a causal role (the exposure slot) in our event template, and none of these health issues had microbial etiology. It definitely takes more consideration for reclassifying and modeling these abusive behaviors (affecting self or others) as exposures.

### 3.4. Limitations

Although we believe the high-level trends to be reliable, the PubMed-based coverage evaluation might involve some marginal errors inevitably. Only discharge summary was utilized in this study, which does not represent all types of clinical text. As an exploratory evaluation, the exposome annotations were curated by only one annotator (JF) and therefore not free from bias. With the aim of just assessing what exposome concepts exist, we did not engage in annotating any advanced attributes such as intensity (dosage), duration, and frequency. Relatedly, we did not attempt to optimize the NLP methods for extracting exposome concepts; instead, we concentrated on understanding the high-level distribution and characteristics of the final annotations.

### 3.5. Future Work

We will consider seeking collaboration with the ExO development team to unify the solutions and make it a sustainable resource for the exposome science community. Beyond serving the coverage evaluation, we will build a knowledge base of clinical exposome by refining our PubMed query and postprocessing pipeline. For rigorous annotation, multiple annotators will be used and with agreement metrics computed. The annotation criteria are to be expanded by including pertinent attributes (e.g., exposure intensity) and documented in a formal guideline. The annotations can be used to train NLP systems for cohort identification in clinical exposome studies. Ultimately, a more thorough expotyping approach should be pursued to integrate both structured and unstructured EHR data.

## 4. Conclusions

We developed a semiautomated approach for modeling exposome semantics and assessing the usability in clinical contexts. The modeling leveraged event templates to identify exposure concepts that had ontological relations with disease outcomes. A subnetwork was derived from the UMLS, representing 454 pairs of relations between exposure and outcome semantic types. The subnetwork was able to cover 90% of PubMed literature on exposure-induced iatrogenic diseases from 2001 to 2010. For identifying exposome concepts in 50 discharge summaries, the subnetwork improved the efficiency by filtering out 78% of irrelevant machine annotations. The exposome concepts exhibited diverse presence of semantic types and sections in the annotated corpus. Analysis into the results expanded our understanding of the clinical narratives and ontology resources. This work demonstrated the value of semantics-powered methods for advancing exposome science and precision medicine.

## Supplementary Material

Supplement 1. Full set of the exposome semantic types and relations. Supplement 2. Full set of the ontology-identified microbial disorder concepts. Supplement 3. Exposome annotation counts ranked by section and semantic type.

## Figures and Tables

**Table 1:** Example disease concepts of microbial etiology.

Disease CUI	Disease name	Disease semantic type	Microbe CUI	Microbe name	Microbe semantic type
C0006057	Botulism	T037 Injury or Poisoning	C0009055	*Clostridium botulinum*	T007 Bacterium
C0275677	Miscarriage due to *Leptospira*	T046 Pathologic Function	T0023358	*Leptospira*	T007 Bacterium
C0376618	Endotoxemia	T033 Finding	C0018150	Gram-negative bacteria	T007 Bacterium
C0266024	Moon's molar teeth	T019 Congenital Abnormality	C0040840	*Treponema pallidum*	T007 Bacterium
^∗^C0032768	Postherpetic neuralgia	T047 Disease or Syndrome	C0042338	Human herpesvirus 3	T005 Virus
^∗^C0032371	Poliomyelitis	T047 Disease or Syndrome	C0206435	Human poliovirus	T005 Virus
^∗^C1535939	*Pneumocystis jiroveci* pneumonia	T047 Disease or Syndrome	C0320385	*Pneumocystis jiroveci*	T004 Fungus
^∗^C0029307	Oroya Fever	T047 Disease or Syndrome	C0318324	*Bartonella bacilliformis*	T007 Bacterium

^∗^Concepts that did occur in the study corpus.

**Table 2:** Example exposure-disease MeSH pairs (and their semantic types) covered by our exposome subnetwork.

PMID	Exposure MeSH	Exposure semantic type	Disease MeSH	Disease semantic type
20736205	Anti-Arrhythmia Agents	T121 Pharmacologic Substance	Hallucinations	T048 Mental or Behavioral Dysfunction
11337626	Osteotomy	T061 Therapeutic or Preventive Procedure	Kyphosis	T190 Anatomical Abnormality
11387778	Colonoscopy	T060 Diagnostic Procedure	Intestinal Perforation	T047 Disease or Syndrome
11581058	Prostheses and Implants	T074 Medical Device	Lacrimal Duct Obstruction	T190 Anatomical Abnormality
11747288	HIV Protease Inhibitors	T121 Pharmacologic Substance	Lipodystrophy	T047 Disease or Syndrome
11984961	Dental Restoration, Permanent	T122 Biomedical or Dental Material	Systemic Inflammatory Response Syndrome	T047 Disease or Syndrome
12043843	Haloperidol	T109 Organic Chemical T121 Pharmacologic Substance	Basal Ganglia Diseases	T047 Disease or Syndrome
12106934	Cardiac Catheterization	T058 Health Care Activity	Arteriovenous Fistula	T190 Anatomical Abnormality

**Table 3:** Top 20 sections with exposure annotations in the 50 Partners discharge summaries.

Rank	Section name	# annotations
1	HOSPITAL COURSE	617
2	HISTORY OF PRESENT ILLNESS	418
3	DISCHARGE MEDICATIONS	344
4	MEDICATIONS ON ADMISSION	321
5	MEDICATIONS ON DISCHARGE	309
6	MEDICATIONS	178
7	PAST MEDICAL HISTORY	89
8	SOCIAL HISTORY	87
9	ALLERGIES	47
10	MEDICATIONS ON TRANSFER	45
11	HOSPITAL COURSE BY SYSTEM	40
12	ADMISSION MEDICATIONS	40
13	RELEVANT LABORATORY DATA	38
14	LABORATORY DATA	36
15	HOSPITAL COURSE AND TREATMENT	35
16	HOSPITALIZATION COURSE	29
17	PHYSICAL EXAMINATION	22
18	PAST SURGICAL HISTORY	22
19	IDENTIFYING DATA	19
20	DISCHARGE INSTRUCTIONS	18

**Table 4:** Top 20 sections with exposure annotations in the 73 Beth discharge summaries.

Rank	Section name	# annotations
1	HOSPITAL COURSE	492
2	HISTORY OF PRESENT ILLNESS	386
3	BRIEF HOSPITAL COURSE	353
4	DISCHARGE MEDICATIONS	267
5	MEDICATIONS ON ADMISSION	157
6	PERTINENT RESULTS	148
7	DISCHARGE INSTRUCTIONS	114
8	DISP	103
9	SOCIAL HISTORY	93
10	PAST MEDICAL HISTORY	92
11	SIGNED ELECTRONICALLY BY	69
12	INDICATION	45
13	THE FOLLOWING ISSUES WERE ADDRESSED DURING THIS HOSPITAL ADMISSION	44
14	GASTROINTESTINAL	44
15	SERVICE	43
16	IMPRESSION	41
17	COURSE IN HOSPITAL	40
18	ALLERGIES	40
19	LABORATORY DATA	38
20	MAJOR SURGICAL OR INVASIVE PROCEDURE	35

**Table 5:** Top 20 semantic types of the exposure annotations in the 50 Partners discharge summaries.

Rank	Semantic type	# annotations
1	T121: Pharmacologic Substance	927
2	T109: Organic Chemical	874
3	T061: Therapeutic or Preventive Procedure	281
4	T060: Diagnostic Procedure	278
5	T195: Antibiotic	105
6	T116: Amino Acid, Peptide, or Protein	72
7	T074: Medical Device	64
8	T033: Finding	47
9	T197: Inorganic Chemical	38
10	T125: Hormone	36
11	T047: Disease or Syndrome	30
12	T200: Clinical Drug	29
13	T127: Vitamin	27
14	T055: Individual Behavior	24
15	T007: Bacterium	15
16	T097: Professional or Occupational Group	14
17	T129: Immunologic Factor	14
18	T114: Nucleic Acid, Nucleoside, or Nucleotide	13
19	T058: Health Care Activity	12
20	T037: Injury or Poisoning	12
